# Future Perspectives in Small-Diameter Vascular Graft Engineering

**DOI:** 10.3390/bioengineering7040160

**Published:** 2020-12-10

**Authors:** Panagiotis Mallis, Alkiviadis Kostakis, Catherine Stavropoulos-Giokas, Efstathios Michalopoulos

**Affiliations:** 1 Hellenic Cord Blood Bank, Biomedical Research Foundation Academy of Athens, 4 Soranou Ephessiou Street, 115 27 Athens, Greece; cstavrop@bioacademy.gr (C.S.-G.); smichal@bioacademy.gr (E.M.); 2 Center of Experimental Surgery and Translational Research, Biomedical Research Foundation Academy of Athens, 4 Soranou Ephessiou Street, 115 27 Athens, Greece; akostakis@bioacademy.gr

**Keywords:** small-diameter vascular grafts, tissue engineering, cardiovascular disease, vascular reconstruction, bypass surgery, decellularization, human umbilical arteries, synthetic materials, 3D and 4D printing, thermoresponsive materials

## Abstract

The increased demands of small-diameter vascular grafts (SDVGs) globally has forced the scientific society to explore alternative strategies utilizing the tissue engineering approaches. Cardiovascular disease (CVD) comprises one of the most lethal groups of non-communicable disorders worldwide. It has been estimated that in Europe, the healthcare cost for the administration of CVD is more than 169 billion €. Common manifestations involve the narrowing or occlusion of blood vessels. The replacement of damaged vessels with autologous grafts represents one of the applied therapeutic approaches in CVD. However, significant drawbacks are accompanying the above procedure; therefore, the exploration of alternative vessel sources must be performed. Engineered SDVGs can be produced through the utilization of non-degradable/degradable and naturally derived materials. Decellularized vessels represent also an alternative valuable source for the development of SDVGs. In this review, a great number of SDVG engineering approaches will be highlighted. Importantly, the state-of-the-art methodologies, which are currently employed, will be comprehensively presented. A discussion summarizing the key marks and the future perspectives of SDVG engineering will be included in this review. Taking into consideration the increased number of patients with CVD, SDVG engineering may assist significantly in cardiovascular reconstructive surgery and, therefore, the overall improvement of patients’ life.

## 1. Introduction

Small-diameter vascular grafts (SDVGs) with inner lumen diameter (d) less than 6 mm are required in vascular reconstructive surgery. Tissue engineering (TE) represents an emerging research field where the production of vascular grafts utilizing state-of-the-art manufacturing methods has gained great attention from the scientific society [1,2]. In contrast to large (d > 8 mm) and medium (d = 6–8 mm) diameter vascular grafts, which have currently been applied in a wide variety of vascular applications, such as carotid and aorta replacement, the production of SDVGs (d < 6 mm) requires further improvement [1,2,3]. Indeed, synthetic vascular grafts, derived from expanded polytetrafluoroethylene (ePTFE) and Dacron, serving as medium- or large-diameter vessel transplants, have shown interesting results in reconstructive surgery [4]. Long-term results of large diameter vascular grafts (LDVGs), e.g., when applied as aortoiliac substitutes, have exhibited good patency rates (90%) within the first year of implantation [2,5,6]. Additionally, medium-diameter vascular grafts, such as the carotid substitutes, are characterized by patency rates greater than 60% after the 1st year of implantation [2,7]. On the other hand, the proper production and use of small-diameter vascular grafts in reconstructive surgery are still under evaluation.

SDVGs are initially aimed to be used in coronary artery bypass grafting (CABG), issued by manifestations of cardiovascular disease (CVD). Regarding non-communicable diseases, CVD is the most leading cause of death globally [8,9]. CVD is a group of complex disorders, including peripheral arterial disease (PAD), coronary heart disease (CHD), cerebrovascular disease, and rheumatic heart disease [8,10]. It has been estimated that in the European Union (EU), CVD causes more than 3.9 million deaths, which accounts for 45% of all deaths each year [11]. Moreover, 11.3 million new cases of CVD are reported in the EU annually [12,13]. Furthermore, the United States is characterized by an increased percentage of CVD cases and deaths [14,15]. It is estimated that more than 400,000 CABG procedures are performed in the USA annually [14,16]. The CVD occurrence is mostly related to changes in dietary habits, reduced exercise, increased working time, depression, national health care deficiencies and the occurred financial crisis [17,18,19,20]. In terms of economic burden, it has been estimated that in Greece, the mean annual healthcare cost per patient is 5495 €, 4594 €, and 8693 € for CHD, CVD, and PAD, respectively [21]. Therefore, the proper development and clinical utilization of functional SDVGs is of paramount importance.

Nowadays, a great number of treatments can be effectively applied in CVD. These treatments may include the change of dietary–lifestyle habits or the application of pharmaceutical and surgical approaches. In the context of vascular surgery intervention, endovascular approaches such as angioplasty, atherectomy, and stent insertion can be performed. Additionally, vascular graft transplantation may be applied as an alternative option to replace or bypass the injured vessels.

To date, the gold standard procedure for CABG is the use of autologous vessels, such as the internal thoracic artery, radial artery, and saphenous vein [22]. Among them, the saphenous vein (SV) is the most widely used graft in SDVGs replacement [23,24,25,26,27]. The first use of saphenous vein in the clinical setting has been reported in 1951 by Kunlin and his colleagues [28]. The SV is characterized by greater patency rates (90% after the 1st year of implantation), compared to synthetic grafts (>60%, within the first year) [7,29,30]. However, significant drawbacks also accompany the use of autologous vessels. It is estimated that >30% of patients with CVD lack suitable vessels [1,31]. Moreover, in the case of the performance of second bypass surgery, the possibility of finding another suitable vessel decreases dramatically [32]. The latter can be performed within 10 years after the initial implantation, considering that the patency rate of autologous vessels (saphenous vein) after the 5 years is approximately less than 50% [2]. Moreover, biomechanical incompliance between arteries and veins can result in neointima formation, immune system activation, and finally graft failure and rejection [32].

Taking into account the above information regarding the use of SDVGs for bypass surgeries, alternative strategies for the development of vessel conduits must be evaluated and established. Tissue engineering may assist significantly to this issue by providing evidence and new ideas for the manufacturing of suitable SDVGs, which will be capable for cell homing, growth, and differentiation, and also characterized by improved in vitro and in vivo remodeling properties. In this review, we will highlight the state-of-the-art methodologies, while the future perspectives of SDVGs will be presented in detail.

## 2. Characteristics of Engineered SDVGs

The manufacturing of SDVGs with the TE methodologies has been improved significantly since the first attempts for production and application of synthetic vascular grafts used in bypass surgeries in the late 1980s [33]. Several years later, the first commercially available tissue-engineered vascular grafts (TEVGs) appeared, including Synergraft^®^ (CryoLife, Inc., Kennesaw, GA, USA), Artegraft^®^ (LeMaitre Vascular, Inc., Burlington, MA, USA), Procol^®^ (LeMaitre Vascular, Inc., Burligton, MA, USA), and Cryovein^®^ (CryoLife, Inc., Kennesaw, GA, USA) [34]. The majority of these grafts have received approval from the Food and Drug Administration (FDA) and the European Medicinal Agency (EMA) for human applications.

The proper design of the vascular grafts ensures successful cell seeding at the pre- and post-implantation stage. Cellular populations may positively influence the vessel graft functionality [35]. The most applied cellular populations are the endothelial cells (ECs) and vascular smooth muscle cells (VSMCs) [36]. ECs are located in the internal layer of the vascular wall, known as tunica intima, forming the endothelium [37]. The endothelium has unique anti-thrombogenic properties, avoiding the platelet aggregations and clots formation [38]. VSMCs are responsible for vasoconstriction and vasodilation, located in the media layer of the vessel wall, which is known as tunica media [39]. Dependent on microenvironment stimuli, the ECs can elevate the levels of endothelial nitric oxide synthetase (eNOS), leading to NO production, which downstream induces the VSMCs-dependent vasodilation [38]. Importantly, VSMCs also support the vascular remodeling and regeneration with the production of extracellular matrix (ECM) proteins such as collagen and elastin [39]. Besides, the beneficial effects of the cellular populations may occur to the vascular graft, their successfully seeding and proliferation may require long-term cultivation periods. Additionally, the isolation and expansion of specialized cellular populations from patients with CVD is a demanding challenge [40]. To date, there is a tendency for developing readily available acellular vascular scaffolds with improved anti-thrombogenic properties [41,42,43,44]. Indeed, these pioneering studies are focusing on the fabrication of a negatively charged synthetic surface in order to avoid red blood cells and platelet aggregation. In this way, the SDVGs must satisfy certain design criteria to be clinically available [45]. Specifically, SDVGs must have similar biomechanical properties (burst pressure, high-stress deformation, and suture strength) with the substituted vessels to avoid aneurysm and neointima development [46]. In addition, regardless of the vascular graft material, engineered vessels must be non-cytotoxic and support cell growth [45]. Engineered SDVGs must be characterized by specific ultrastructure, ensuring the cell seeding, proliferation, and differentiation [2]. Finally, the engineered SDVGs must not be immunogenic, and also must be characterized by in vivo remodeling and regeneration properties [2].

Nowadays, a wide variety of manufacturing techniques for SDVGs such as the use of synthetic polymers, decellularized natural matrices, bioprinting, and 4D printing have been developed, although the majority of them require further evaluation and optimization.

## 3. TEVGs Derived from Synthetic Polymers

**Figure 1:**
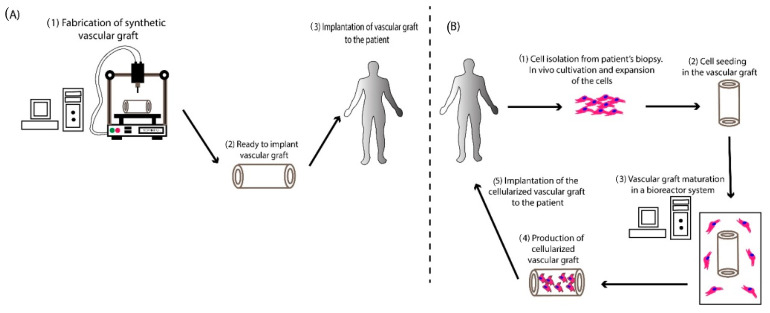
Development and implantation of engineered small-diameter vascular grafts (SDVGs). (**A**) The first approach comprises the production of acellular SDVG derived from polymer materials using the state-of-art bioprinting approach. Then, the manufactured SDVG can be implanted immediately into the patient. In this approach, the patient’s body will serve as a bioreactor for the recellularization of the implanted vascular graft. However, some major disadvantages, including the time period needed for the proper cellularization or the impaired functionality of the produced vascular grafts, maybe existed. (**B**) The second approach comprises the combination of cellular populations with the polymer derived SDVGs. In this approach, the cells can be isolated from the patient’s tissue biopsy, in vitro expanded, and seeded onto the SDVG. Finally, the engineered SDVG can be implanted back to the patient. The advantage of this approach is the production of compatible SDVGs with the patients, avoiding in this way any potential adverse reactions.

Manufactured TEVGs from polymer materials have been widely used in vascular reconstructive surgery in the last years [47,48]. The use of synthetic polymers has brought a new era in surgery, decreasing the time needed for vessel conduit manufacturing. Vascular grafts produced from synthetic materials can be manufactured with state-of-the-art tissue engineering methods, including tissue engineering by self-assembly (TESA), electrospinning, and bioprinting. Among them, bioprinting has gained great attention from the scientific society due to the production of high-quality tissue engineering vascular scaffolds. The manufactured scaffolds (acellular or cellularized conduits) can be implanted in the patient to replace the damaged vessels (Figure 1). Synthetic conduits can be divided into non-degradable, degradable polymers, and biopolymers. Each category is characterized by specific characteristics, which will be further explored in this review article.

### 3.1. Non-Degradable Polymers

Non-degradable polymers were among the first materials used as a source for the production of vascular grafts that have been employed in bypass surgeries (Table 1). Historically, the first attempt for the production of ePTFE material has been performed by Robert Gore in 1969 [4]. Several years later, Campbell et al. reported promising results regarding the use of ePTFE vascular grafts in 15 patients as a femoropopliteal bypass graft [49]. In 1986, Weinberg and Bell [33] developed the first tissue-engineered blood vessel substitute through culturing of bovine ECs, VSMCs, and fibroblasts to a Dacron derived conduit. Since then, a great effort by the research teams has been performed establishing new strategies to obtain functional TEVGs. EPTFE, Dacron, and polyurethanes are the most used materials for the production of non-degradable vessel conduits [48]. Compared to autologous vessels, synthetic non-degradable conduits are characterized by a lower percentage of patency rates when used as SDVGs [50]. To date, Dacron is preferred to be used as a material for the production of vessel conduits due to improved biomechanical properties [48,51]. However, both of them exhibit significant adverse reactions. Specifically, a generalized immune response toward the polymers is exerted mostly by macrophages and T cells [52,53]. This could lead to lumen occlusion, which may be treated with new cardiovascular reconstructive surgery. Moreover, most of these grafts lack arginine-glycine-aspartic acid (RGD) binding sites in order to promote cell adhesion [54]. In this direction, several alternative strategies have been employed such as the chemical modification or pre-coating of the polymer materials toward favoring the cell adhesion. Indeed, the addition of P15 peptide, pre-coating with fibronectin, or cross-linked RGD binding sites have been suggested as alternative strategies for improving ECs and VSMCs seeding on polymer scaffolds [55]. A number of research groups have performed pre-coating of polymer vascular grafts with fibroblast growth factor (FGF), vascular endothelial growth factor (VEGF), and epidermal growth factor (EGF), improving in this way the ECs, VSMCs, and fibroblasts mobilization, seeding, and proliferation onto the produced graft [56,57,58]. Randone et al. [59] reported the efficient production of VEGF pre-coated ePTFE vascular grafts. The results of this study showed increased ECs proliferation and endothelium formation in VEGF pre-coated grafts compared to non-pre-coated vascular grafts. In addition, Randone et al. reported that the microporous structure of ePTFE was ideal for ECs seeding, thus grafts with high porosity (>90 μm) may have better endothelialization outcomes [59]. It is known that VEGF exerts chemoattractant and mitogenic abilities on ECs. In this way, the ECs can be attracted by the VEGF pre-coated graft [60]. During ECs proliferation, a significant amount of growth factors are released, which can further regulate the function of vessel resident cellular populations, such as the VSMCs and the fibroblasts [61].

Another important issue that should be addressed is the possibility of thrombus formation. Typically, the polymer acellular vascular grafts are preferred mostly due to the short manufacturing time that is needed. On the other hand, the absence of an organized endothelium could result in increased platelet aggregation and thrombus formation [62,63]. This series of events can cause serious adverse events to the patients that might be even life-threatening. A possible solution to this issue could be the production of polymers with anti-thrombogenic surface or polymers with the substantial release of anti-thrombogenic molecules. Hoshi et al. [64] have reported the successful production of heparin-modified ePTFE vascular grafts. Moreover, Hoshi et al. managed to develop an easily implemented approach, including the covalent link of heparin to the inner side of the ePTFE grafts, to produce vascular grafts with anti-thrombogenic properties [64]. The produced graft inhibited successfully the platelet adhesion; however, a minor negative effect in endothelial cell function was evident. Furthermore, heparin-modified ePTFE vascular grafts were characterized by the high stability of their modified surface area, which was retained for a long time period (28 days) [64]. Moreover, it should be noted that non-degradable polymers are characterized by specific biomechanical properties. Mismatch of tubular compliance may exist in vascular grafts derived from non-degradable polymers. This phenomenon is mostly occurred due to the pre-existing differences in elasticity between the TEVG and the native artery. It is known that small diameter arteries, which are characterized by specific mechanical properties, can absorb energy (pulsatile energy) during the vasoconstriction, which is further released during vasodilation, contributing to the pulsatile blood flow. In this way, a vascular graft, which is characterized by a stiffer behavior than the native ones, can diminish the pulsatile energy by 60%. This compliance mismatch between the two vessels can lead to intima hyperplasia, immune system overactivation, and final graft failure.

### 3.2. Degradable Polymers

Degradable polymers can be used as an alternative strategy for the production of SDVGs (Table 2). These materials can be substantially degraded, forming a proper ECM [47]. Hydrolysis of the ester bonds of the scaffolds and the metabolism of polymers into H_2_O and CO_2_ comprises the main degradation mechanism. The most known degradable materials are the poly (lactide-co-glycolide) (PLGA), polyglycolic acid (PGA), poly-lactic acid (PLA), poly-l-lactic acid (PLLA), polyglycerol sebacate (PGS), and polycaprolactone (PC) [47,48]. The above materials have been extensively used for the production of TEVGs with large and medium lumen diameter. Currently, these polymers have been proposed as starting materials for the production of SDVGs, while their efficient in vivo application is still under evaluation. Each material is characterized by unique properties. Indeed, the molecular structure, the polymerization transition temperature, and biomechanical behavior are some of the different properties that may exist among the materials [73]. For instance, PGA is characterized by rapid degradation time, which affects its biomechanical properties [47,48]. For this purpose, the degradation time can be controlled through polymerization with other materials such as PLA. PGS, another material that is used for the fabrication of TEVGs, can be fully degraded within 30 days [47,48]. PLA is a material whose complete degradation may last over years [2]. This material is characterized by a stiffer behavior than the PGA and also by improved endothelialization and patency rates. PCL is a hydrophobic material with long-term degradation time and, due to this, can sustain better initial biomechanical properties [47,48]. The first report regarding the biocompatibility and biodegradability of the polymer materials was performed in 1966 by Kulkarni and his colleagues [74]. Specifically, it was shown that PLA does not bear any cytotoxic factors and could be used in various applications, such as the production of surgical implants, without causing any tissue reaction.

Degradable polymers represent a valuable source for the production of acellular large, medium, and small diameter vessel conduits, reducing the manufacturing time even more. On the other hand, significant adverse reactions have been reported regarding their use. One major drawback is the lack of RGD-binding motifs, leading to ineffective cell seeding and proliferation [75]. As a consequence, organized endothelium cannot be formed, which can result in platelet aggregation, clot formation, and lumen occlusion [43]. For this purpose, several research groups are evaluating novel strategies for the efficient endothelialization of the luminal surface of the polymer-derived vascular grafts [2,47,48,76]. Previous strategies including chemical modifications and lumen surface pre-coating have also been employed to scaffolds derived from degradable polymers to improve further their functionality. Wang et al. [77] managed to develop an SDVG using a combination of PCL and gelatin. In addition, surface modification with heparin was also performed [77]. The produced vascular conduits were implanted in rats as an abdominal artery graft and remained patent for 12 weeks [77]. These grafts were proven capable of efficient recellularization by ECs. In the same way, Quint et al. [78] used a PGA vascular graft as a scaffold for in vitro recellularization with aortic SMCs. Then, these grafts were placed in a pulsatile bioreactor system for 10 weeks followed by decellularization [78]. The occurred acellular vascular graft was reseeded with ECs and endothelial progenitor cells (EPCs) in order to avoid thrombus formation. Finally, the vascular conduit was implanted to a porcine model as a common carotid artery interposition graft and remained for 30 days [78]. The results of this study showed the efficient production of a personalized vascular graft, which has retained its ability for in vivo remodeling [78]. To date, a small number of clinical trials with degradable SDVGs have been performed (Table 2). Specifically, Lawson et al. [79] developed PGA-based SDVGs that were initially repopulated with VSMCs in a bioreactor setting. Then, pulsatile cyclic distension for 8 weeks, was applied to the SDVGs, followed by decellurization procedure. The occurred acellular SDVGs were applied as an arteriovenous graft in 60 patients (divided into two studies). In both studies, the average primary patency rate was 58% and 23%, after 6 and 12 months, respectively [79]. No aneurysm formation or immune response against the SDVGs was observed in all patients. In total, 4 patients died from end-stage renal disease (ESRD) manifestations rather than vascular graft complications. Moreover, histological analysis in SDVGs segments after 16 weeks of implantation showed infiltration by CD68^+^ monocytic cells, SMA^+^ VSMCs, and CD31^+^ ECs [79]. On the contrary, no T or B cells were evident in the histological analysis. The above outcome is quite promising, widening in this way the clinical feasibility of degradable SDVGs.

### 3.3. Biopolymers

Besides the use of non-degradable and degradable vascular grafts, conduits based on natural matrices and proteins have also been proposed as an alternative solution (Table 3) [1]. These proteins can be used as the structural basis for the development of SDVGs, providing an appealing 3D microenvironment with proper binding sites for the cellular populations [47,48]. Several methods have been proposed to properly produce biopolymer-based SDVGs, including electrospinning, freeze-drying, and mold casting.

Collagen and its isoforms are the most abundant proteins that can be easily isolated, manipulated, and used for scaffold production, including also the engineered SDVGs. Habermehl et al. [89] optimized the procedure for collagen isolation from rat tails, and since then, a wide number of applications, where this structural protein is the main player, have been reported [90,91,92,93]. Until now, 28 different collagen types have been reported [94]. The collagen structure is composed of a repeated triple helix of proline (X) and hydroxyproline (Y). Based on the triple helix organization, collagen can be distinguished into a) fibrils (including types I-V and XI), networks (including types IV-X), and filaments (including type VI) [94]. Among them, collagen I is the most abundant type in mammalians, composed of two α_1_(Ι) and one α_2_(Ι) chains. Collagen type I offers a great number of integrin-binding sites, which can control the cell adhesion, differentiation, and overall cellular behavior. Different types of collagen-based scaffolds have been used in tissue engineering applications [91]. Collagen scaffolds combined with hydroxyapatite have been used in orthopedic applications, inducing bone and cartilage regeneration [95,96]. Moreover, collagen has been proposed as a drug delivery system (DDS) to release pro-angiogenic factors for wound healing applications and as a natural coating of vascular grafts [97,98]. However, collagen is characterized by low mechanical properties and increased thrombogenicity [99,100]. For this purpose, cross-linking with fixative agents such as glutaldeyhyde has been proposed [101,102]. Nevertheless, the improvement in mechanical properties, severe cytotoxicity are accompanied most of the time due to the crosslinking agent that was applied [103]. Alternative crosslinking methods have also been utilized such as photo-crosslinking or the use of carbodiimide [104,105,106]. Moreover, collagen-based SDVGs combined with fibronectin or elastin fibers have shown promising results regarding the biomechanical and anti-thrombogenic properties. Another promising biomaterial for SDVGs fabrication is the silk fibroin [107,108]. Fibroin is derived from Bombyx mori (silkworm) and is composed of β-sheet crystal and semicrystalline regions occurred after the removal of sericin [109,110]. Sericin is a highly antigenic protein, which covers the silk fibers [111]. Additionally, fibroin has anti-thrombogenic properties and can be degraded over time, therefore, could be a valuable source for the production of SDVGs [112]. Enomoto et al. [113] managed to develop a fibroin-based SDVG whose patency was compared with ePTFE vessel conduits. In this study, the developed conduits (d = 1.5 mm, l = 10 mm) were implanted as abdominal aorta interposition grafts in male Sprague-Dawley rats for a time period of 72 weeks [113]. Fibroin based SDVGs remained patent (85%) over 64 weeks, whereas ePTFE grafts were patent (48%) for 32 weeks [113]. In addition, an increased number of SMCs and ECs was observed in fibroin-based SDVGs compared to ePTFE grafts, reflecting in this way the impaired overall functionality of the latter.

To date, fibrin, which can be obtained from peripheral blood, comprises a biomaterial that can be applied in SDVG engineering [114,115]. Fibrin is produced through the cleavage of fibrinogen [115]. Fibrinogen (MW: 340 kDa), a glycoprotein that is abundant in plasma, contains three pairs of polypeptide chains, the Aα, Bβ, and γ, which are connected with 29 disulfide bonds [115]. Upon stimulation, thrombin cleaves the fibrinopeptides A and B, between Arg-Gly residues [115,116]. The remained (α, β, γ)_2_ can be polymerized with other fibrin molecules, resulting in the production of fibrin final form. Due to that, fibrin can be produced from patients’ blood, without causing any negative adverse reactions to the recipient [115,116]. Fibrin is a rich source of growth factors, cytokines, and chemokines, such as Tumor Necrosis Factor-A (TNF-A), Vascular Endothelial Growth Factor (VEGF), Fibroblast Growth Factor (FGF), Platelet-Derived Growth Factor AA (PDGF-AA), Interleukin 1A (IL-1A), IL-1B, IL-2, IL-6, IL-8, TNF-Receptor type-1 associated Death domain protein (TRADD), CC-motif chemokine receptor 1, etc. [117,118]. Recently, platelet-rich plasma or fibrin gel have been employed in a series of regenerative medicine applications such as skin wound healing and dystrophic recessive epidermolysis bullosa [119,120]. Except for the patient’s own blood, fibrin can be produced from other sources like the umbilical cord blood (UCB). Rebulla et al. [121] initially optimized the PRP and fibrin production from UCB units that did not meet the criteria for cryopreservation. In addition, our group suggested a protocol for the efficient production of PRP and fibrin from low volume CBU units [118]. The development of allogeneic fibrin holds significant advantages such as the avoidance of repeating blood sampling, especially from severe conditioned or elderly individuals, low immunogenicity of the obtained fibrin, and absence of allergic reaction [118].

Recently, fibrin has been employed in vascular tissue engineering. In the beginning, fibrin was used as a coating in collagen-based vascular grafts [122]. To date, the research society is performing a significant effort to produce fibrin-based vascular grafts [123,124]. Most of the time, a pulsatile bioreactor system is required for the proper maturation of the developed vascular grafts. Moreover, approaches, where ECs and SMCs are utilized in fibrin-based vascular grafts, have been proposed [36]. Swartz et al. [125] used recellularized fibrin-based vascular grafts as implants in a sheep model. Specifically, these grafts were implanted in the jugular veins for a time period of 15 weeks. Histological analysis of the grafts showed the successful in vivo remodeling, where collagen and elastin depositions were evident [125]. However, the fibrin-based vascular grafts were characterized by impaired biomechanical properties. Indeed, the average burst pressure of fibrin-based vascular grafts was 543 ± 77 mmHg, which is very low to withstand the physiological burst pressures of blood flow [125]. A recent study from Yang et al. [126] showed that the mechanical properties of these vessel conduits can be improved with the addition of PCL, resulting in the production of a hybrid graft (fibrin-PCL vascular graft). In this study, electrospun PCL/fibrin vascular grafts were developed, followed by evaluation of mechanical properties, cytotoxic effects, and in vivo biocompatibility [126]. The burst pressure of these hybrid vascular grafts was 1811 ± 101 mmHg, which is similar to native blood vessels (2000 mmHg). Furthermore, no cytotoxic effects or in vivo immune response were reported, in this study [126].

The production of vascular grafts made of chitosan has also been reported [127]. Chitosan is a linear polysaccharide that is closely related to sulfated glycosaminoglycans (sGAGs) [128]. Chitosan is a natural material that is derived from the shell of shrimps and crabs and has been used extensively in a wide range of tissue engineering applications [128]. Specifically, chitosan has been used for the production of hydrogels, DDS, coatings, and also in wound healing applications [128]. In addition, chitosan can be combined with degradable polymers such as PCL and PLA for scaffold fabrication [129]. Moreover, chitosan has mild antibacterial properties, which are beneficial for in vivo applications [130]. Recently, the use of chitosan has been proposed for the development of SDVGs. In the context of vascular graft production, the electrospinning technology can be utilized to produce conduits with wide pore distribution, high porosity, and adequate microenvironment for cell adhesion and proliferation. Wang et al. [127] reported the development of a PCL/chitosan (PCL/Ch) hybrid-based SDVG with anti-thrombogenic and anti-bacterial properties. For the scaffold fabrication, the electrospinning technology was utilized [127]. The results of this study showed that the PCL/Ch hybrid-based SDVGs have similar anti-thrombogenic properties as the heparin-coated vessel conduits, while the bacterial killing ratios were 64% for *S. aureus* and 73% *E. coli* [127]. Yao et al. [129] also developed electrospun PCL/Ch SDVGs, which were further combined with heparin and referred as Hep-PC/Ch grafts. These grafts were further implanted as aortic replacements in male Sprague-Dawley rats. Their functionality was compared with PCL/Ch vascular grafts (without heparin immobilization). After 4 weeks of implantation in rats, the PCL/Ch explants were characterized by thrombus formation, while no thrombus formation was observed to Hep-PCL/Ch grafts [129]. Furthermore, Hep-PCL/Ch grafts were characterized by good patency rate and successful endothelialization as were indicated by SEM analysis [129]. Taking into consideration the above data, chitosan is a material that can be used in combination with degradable polymers to produce functional SDVGs.

### 3.4. Hybrid Polymers

The proper combination of synthetic and natural polymers could produce functional engineered SDVGs. These conduits combine the beneficial features of both materials and are characterized by improved biomechanical, anti-thrombogenic, anti-bacterial, and cell adhesion properties [139]. Furthermore, hybrid vascular grafts can be combined with key specific growth factors such as TGF-β1, VEGF, EGF, HGF, etc., which can be accumulated in the vascular wall [129,140,141]. These growth factors can be spatially released from there, affecting in this way several cellular functions including cell migration and growth [142]. To date, there is an increasing number of research teams, which are focusing on the production of hybrid vessel conduits (Table 4). Tillman et al. [143] produced a PCL/collagen vascular graft, with improved biomechanical properties. The PCL/collagen conduits served as the aorta and iliac artery interposition grafts in rabbits and remained for a time period of 1 month [143]. These hybrid grafts were free of any aneurism or thrombus formation, while Doppler ultrasound showed good patency (85%) of the grafts. Histological analysis of the explants revealed the absence of inflammation, thus completely lacking any infiltrating immune cell [143]. Wise et al. [144] produced PCL/elastin vascular grafts, where parameters such as ECs adhesion and proliferation, blood biocompatibility, burst pressure, and in vivo functionality were assessed [144]. Specifically, these grafts were able to be recellularize both in vitro and in vivo with the ECs. The burst pressure of the grafts was 1500 ± 150 mmHg; however, it was less than the minimum burst pressure that was evident in human native blood vessels (1700 mmHg). Similar good patency and cell infiltration results of hybrid acellular vascular grafts have been reported in the literature [144,145,146,147].

In addition to these fabrication strategies, the use of cellularized hybrid vascular grafts may provide better outcomes regarding the mechanical properties and overall patency [34]. Thomas and Nair [148] developed a vascular graft, which was composed of gelatin/vinyl acetate copolymers, utilizing the electrospinning method. The composed vascular grafts were successfully recellularized with murine SMCs, followed by maturation with a pulsatile bioreactor system [148]. The pulsatile forces, generated by the bioreactor, effectively stimulated the SMCs migration, proliferation, and gene and protein expression [149].

The overwhelming increase of new CVD cases each year is leading to the exploration of alternative sources for the production of engineered SDVGs. Most of these approaches, including non-degradable, degradable, and biopolymer grafts, are requiring extended evaluation, while their proper fabrication could last over 28 days [2,34]. Toward these shortcomings, the hybrid-based TEVGs may pose a reliable approach, reducing the manufacturing time and thus producing SDVGs with improved properties. However, more research is needed to be performed in order for the hybrid SDVGs to be readily used by clinicians in cardiovascular reconstructive surgery.

## 4. Decellularized Vascular Grafts

In the last decade, the application of the decellularization method for the production of vascular grafts has gained significant attention from the scientific society [154]. Decellularization aims to remove completely the cellular material from the tissue while preserving the ultrastructure of ECM. Depending on the tissue source, different decellularization approaches may be applied to produce effectively an acellular matrix [154,155]. Until now, decellularization has been applied successfully to a great number of organs and tissues, including lung, liver, kidney, heart, cartilage, etc.

### 4.1. Decellularization as a Method for the Production of Vascular Grafts

To achieve the production of an acellular matrix, different decellularization protocols may be used. Mostly, the decellularization protocols include physical, chemical, enzymatic, or a combination of those methods to acquire the best outcome [154,155,156]. The initial step of the decellularization approach is cell destruction through the solubilization of the cytoplasmic membrane and DNA fragmentation. Then, the cellular and nuclear debris must be completely removed from the tissue’s ECM. Excessive removal of decellularization solutions also is an important step of the process to limit the possibility of any cytotoxic effects [154,155,156]. The final step of the decellularization procedure is the sterilization of the produced scaffold.

Sterilization can be achieved either by immersion of the scaffold into antibiotic solutions or by applying physical methods such as UV and γ-irradiation [155,156].

The increased global demand for vascular grafts led the researchers to evaluate further the decellularization approach for the production of vessel conduits [36]. Large- and small-diameter vascular grafts have been decellularized with the application of different decellularization approaches. Mostly, a proper combination of the decellularization approaches, such as snap freezing, use of ionic and non-ionic detergents, trypsin addition, and mechanical agitation or sonication, have been utilized [154,156]. Among them, the use of chemical compounds in combination with physical methods, is the most effective and safe for producing acellular vascular grafts. The most used detergents for the decellularization procedure are sodium dodecyl sulfate (SDS), sodium deoxycholate (SD), Triton X-100, Triton X-200, 3-[(3-Cholamidopropyl)dimethylammonio]-1- propane sulfonate (CHAPS), and Ethylenediaminetetraacetic acid (EDTA) [156]. Additionally, in the literature, the combination of hypotonic and hypertonic treatments, with enzymatic digestion, has been also reported for the efficient production of acellular SDVGs [154,155,156].

**Figure 2:**
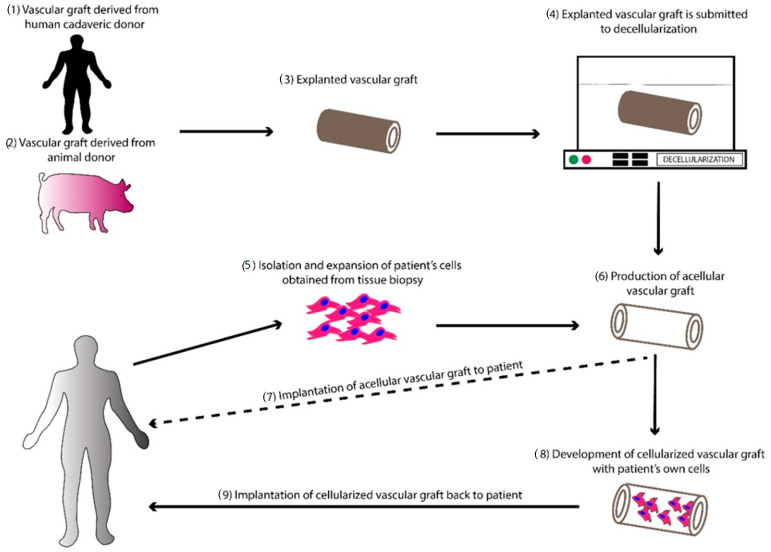
Production of SDVGs with the decellularization approach. Initially, vascular grafts can be obtained either from human cadaveric or animal donor. Then, the obtained SDVG is submitted to decellularization to remove the residual cellular population. The produced acellular vascular graft can be either implanted back to the patient or to submitted in recellularization with the patient’s own cells. The cellular populations can be isolated and expanded from the patient’s tissue biopsy. When cells reached the desired cell number, they can be used for the recellularization of the acellular vascular graft. Finally, the produced cellularized SDVG can be implanted in the patient. The whole procedure can be performed under good manufacturing practice (GMP) conditions.

Taking into consideration the above data, vascular grafts and especially SDVGs can be derived from various sources such as animals (porcine or sheep) or cadaver donors, effectively decellularized, and immediately used (Figure 2). However, significant drawbacks are accompanying the above proposal. In the past, a great effort regarding the use of animal-derived TEVGs in human applications was performed [157]. Despite the complete removal of cellular and nuclear materials from the vessel’s ECM, animal-derived vascular grafts can induce an extended immune response due to the presence of alpha-gal-epitope (Galalpha1-3Galbeta1-(3)4GlcNAc-R) [158]. This epitope is abundant in non-primates and New World monkeys and synthesized by the alpha1,3galactosyltransferase (alpha1,3 GT) [158,159,160]. On the other hand, humans, apes, and Old World monkeys produce anti-Gal antibodies, which are representing 1% of the circulating immunoglobulins [158,159,160]. In this way, human recipients when receiving animal-derived vascular grafts, exert significant immune response against the aforementioned grafts, which finally leads to graft rejection. Nowadays, much effort has been focused on the cleavage of a-gal epitope or the production of transgenic animals (without the presence of a-gal epitope), although more research must be performed toward this direction [161,162]. Recently, genome editing with CRISPR-Cas9 may assist in this field [163]. Cadaver donors may constitute an alternative source, for obtaining SDVGs. Based on organ donation statistics, only 3 in 1000 people find suitable organs and more than 112,000 people are waiting for organ transplantation [164]. Furthermore, the bioethics rules must be modified in order to allow organ transplantation and especially vessel transplantation. The production of vascular grafts with the decellularization approach may be a promising approach, thus increasing the number of available transplantable vessels.

### 4.2. Establishment of the Decellularization Approach

The production of a completely acellular scaffold is a demanding process; however, most of the time a small quantity of residual cellular and nuclear materials are evident. Different decellularization methods are characterized by variable results, indicating that the majority of them cannot produce a completely acellular scaffold. The presence of the cellular components could induce an immune response and hyperacute reaction by the host upon implantation [165,166]. This could lead to unfavorable adverse reactions, resulting in graft occlusion, calcification, and rejection, with the majority of them to be life-threatening for the recipients. Globally, several researchers have tried to validate the decellularization approach in different tissues and organs, leading them to several criteria for the establishment of the successful decellularization approach. Among them, Gilbert et al., Crapo et al., and Badylak et al. have performed the most valuable work, proposing the following criteria [154,156,167].

- <50 ng/double-stranded (ds) DNA/mg ECM dry weight
- <200 bp DNA fragmented length
- Lack of visible nuclear materials, either with 4′,6-diamidino-2-Phenylindole (DAPI) or hematoxylin and eosin (H&E)

Except for the above-mentioned criteria, the total amount of DNA including single-stranded (ss) and ds, should also be quantified and taken into account. DNA quantification can be performed photometrically, or with the use of different commercial kits such as the Picogreen Assay. Indeed, there are numerous studies where Picogreen assay is the optimum method for the quantification of the DNA in decellularized matrices [168,169,170,171]. However, the PicoGreen assay can detect only the ds DNA, while the ss DNA cannot be quantified. On the other hand, the spectrophotometric quantification of DNA by measuring the ratio of absorbance 260 nm/280 nm, may provide more data regarding the presence of the total DNA in the acellular scaffold [172]. It is known that either ss or ds DNA can induce the host’s immune reaction, and the accurate DNA quantification is of major importance. Furthermore, the remaining DNA in the scaffolds can be evaluated through the performance of gel electrophoresis [172]. Typically, the DNA samples can be loaded onto 1–2% *w*/*v* agarose gels and observed under UV light. The absence of dense DNA bands or bands with less than 200 bp DNA confirms further the successful decellularization [172].

The last criterion involves the observation of the tissue sections for any possible nuclear material either with H&E or DAPI [154,156]. H&E is the first-line histological stain that is performed in order to properly evaluate the success of the decellularization approach. The absence of black stain in the histological samples indicates the loss of nuclear material. Besides H&E, more specific stains can be applied for the determination of decellularization. Masson’s trichrome (MT), which stains collagen (blue), muscle cells (red), and nuclear materials (black), can be used for the evaluation of the presence or absence of SMCs. Except for the content of the cellular population, this stain can indicate the proper preservation of the collagen fibers in the acellular scaffold. In the same way, Elastic van Gieson (EVG) can stain simultaneously the elastic/collagen fibers and nuclear material [154,156].

Nevertheless, the production of a completely acellular scaffold is optimum, and the preservation of key ECM features such as the orientation of collagen and elastin fibers are also important. The microarchitecture structure of tissues and organs can determine the decellularization approach, which will be selected. Complex tissues, where the orientation of collagen and elastin determine eventually their biomechanical properties, can be decellularized with the use of non-enzymatic approaches [173,174,175]. It has been shown that trypsin can damage significantly the collagen fibers of a tissue, affecting in this way possibly the graft’s biomechanical properties and cell-binding sites. A balance between the proper elimination of cellular components in combination with the minimum effect in ECM key proteins must be found when a decellularization protocol is applied.

### 4.3. Decellularized Animal-Derived SDVGs

The first attempt for establishing a decellularization protocol was performed in 1966, several years before the attempts of Weinberg and Bell for the manufacturing of synthetic polymer vascular graft [33]. Rosenberg et al. [176] applied for the first time an enzymatic decellularization protocol in bovine carotid arteries. The produced acellular vascular grafts were implanted in 16 patients as femoropopliteal and iliofemoral bypass grafts. The implanted grafts withstood the blood flow pressure; however, graft occlusion was reported during a time period of 2 years postoperatively [176]. Since then, new decellularization approaches have been found and validated in a wide range of tissues and organs including the vessels such as the aorta, carotid, and coronary arteries (Table 5) [177,178,179,180]. The first decellularized vascular grafts were derived from bovine vessels and ureters, which further became commercially available as Artegraft^®^, Solcograft^®^, ProCol^®^ (LeMaitre Vascular, Inc., Burligton, MA, USA), etc. [2,181]. Today, several companies are focused on the production of decellularized vascular conduits based on the bovine vessels. However, the presence of a-gal is a significant limitation, and in order to overcome this issue, crosslinking with fixative agents such as glutaraldehyde is performed [103]. A significant drawback to this approach is the cytotoxicity mediated by the fixative agents, resulting in minimum applicability of those grafts [103]. However, modern fixative agents such as carbodiimide with low or no cytotoxicity have been applied [106]. Another significant drawback of the crosslinked decellularized bovine blood vessels is the lack of in vivo remodeling properties, which makes them unavailable for applications in pediatric patients [182]. Additionally, it has been reported that decellularized animal-derived blood vessels are characterized by similar patency rates as synthetic vascular grafts [36].

Recently, the use of small intestine submucosa (SIS) has been also proposed for the production of large and small TEVGs [183]. Typically, SIS can be derived either from the porcine or ovine origin [184]. Decellularization can efficiently be applied in SIS, and then the produced material can be folded in a tubular mandrel to produce a vascular graft [184]. Moreover, crosslinking with fixative agents such as glutaraldehyde has been reported as an important step in the manufacturing process [185]. These grafts currently have been evaluated for their functionality in animal models, showing good patency rates [36]. Moreover, the patency rates were superior or equal to ePTFE grafts and native ovine artery [36].

Nowadays, the cost production of synthetic vascular grafts has been reduced and, considering the above data, their application is more preferable [36]. On the other hand, due to the increased demand for SDVGs, alternative sources must be explored in order to cardiovascular surgeons to have more available options.

### 4.4. Decellularized Human-Derived SDVGs

The first human blood vessel conduits served as transplants in reconstructive surgery were derived from human cadaver femoral veins. Indeed, human femoral arteries were submitted to decellularization to produce acellular vascular conduits [192]. These grafts were used initially as arteriovenous fistulas (AVF) allografts [193]. Furthermore, these grafts were commercialized under the name Synergraft^®^ (CryoLife, Inc., Kennesaw, GA, USA) [34]. Decellularized iliac vein is another human vascular graft that has been proposed for vascular reconstruction applications [194]. Moreover, this graft was recellularized with the patient’s cells such as ECs and SMCs and then was applied in a 10-year-old female patient with extrahepatic vein obstruction [195]. Before the cell seeding, the graft was evaluated for the presence of cell/nuclear materials and HLA class I and II genes. The operation was performed at Sahlgrenska University Hospital in Gothenburg, Sweden, and the outcomes were published in 2012 [195]. After 1 year of implantation, the graft was occluded, explanted, and a new vein graft was used. Finally, the patient responded well, no anti-endothelial cell antibodies were detected, and there was no need for receiving any immunosuppressive agents [195]. In this direction, human umbilical vessels may be an alternative source for the production of SDVGs [196]. The human umbilical cord (hUC) contains approximately two arteries and one vein, which are mediating in gas exchange and nutrient supply through the fetomaternal circulation [197]. The human umbilical arteries (hUAs) are responsible for the transportation of non-oxygenated blood from the fetus to the mother, while the human umbilical vein (hUV) performs exactly the opposite process [198]. The HUAs and hUVs are characterized by three layers, the inner (tunica intima), the media (tunica media), and the external layer (tunica adventitia). In addition, the hUAs and hUV are vessels without branches and their entire length can be varied and is dependent on hUCs length [197]. The length of a typical hUC is 20–60 cm and is characterized by an average number of 40 helical turns. In addition, hUAs are characterized by specific protrusions located in the tunica intima, throughout the entire vessels, which are known as “Hoboken valves” [199]. These valves prevent successfully the reflux of the non-oxygenated blood back to the fetus. Both vessels can easily and non-invasively be isolated from the hUC after gestation. Typically, in the case of using the human umbilical blood vessels, signed informed consent from the mothers must be obtained [196]. The informed consent should fulfill the requirements of the National law, regarding cord tissue donation and also should be in accordance with the Helsinki declaration.

HUV has been applied as a vascular bypass graft since 1974, followed by commercialization, which was known as Biograft^®^ [200,201]. Several years later, the outcome of the use of Biograft^®^ was evaluated. Specifically, a comprehensive evaluation of the use of hUV as a femoropopliteal bypass graft, a study involved 133 operations and a 5-year follow up, was performed [202]. In this study, it was shown that 6% of the patients died within 30 days after the implantation. The majority of the complications in patients were evident within the first 3 months postoperatively. The mean patency rate was 65% and 50% within the first and fifth year, respectively. No infection of the graft was reported in the current study [202]. The obtained results of the current study indicated that the stabilized hUV could potentially be used as a source for SDVG production. Currently, the gold standard autologous graft for coronary artery bypass surgeries is the SV [203]. However, other blood vessel sources have been evaluated such as the cephalic artery, stabilized hUV, and ePTFE grafts (Table 6). Among them, the hUV seems to share better patency and biocompatibility properties compared to the cephalic and ePTFE vessel conduits [204]. Indeed, a randomized clinical trial has shown that the patency rate of ePTFE was 40% within the first year of implantation, while stabilized hUV was 75% for the same time period [204]. Moreover, SV and stabilized hUV seems to share similar patency rates. Although the results were quite promising, the hUV was stopped to be used as a vessel substitute due to significant drawbacks [204]. HUV is more difficult to be applied technically than SV or synthetic conduits. Moreover, hUV may lack elasticity, making it more fragile [200,201,202,204,205]. In addition, the crosslinking reagents used for its stabilization like glutaraldehyde could induce severe cytotoxicity. Another drawback that is accompanied by the use of the crosslinking agents is the lack of in vivo remodeling properties, which make it less available for pediatric patients [204].

Taking into consideration the above data, the use of hUAs as possible vascular conduits should be also evaluated. Kerdjoudj et al. [206,207] used for the first time the human umbilical artery as potential small-diameter vascular grafts. Initially, this approach involved the deposition of a synthetic polyelectrolyte film in hUAs in order to avoid the platelet adhesion and eventually the thrombus formation [206,207]. In this study, the hUAs were enzymatically de-endothelialized and treated with poly(styrene sulfonate)/poly(allylamine hydrochloride) (PSS/PAH) to develop multilayers of polyelectrolyte film. This negative polyelectrolyte film can exert key anti-thrombogenic properties, avoiding in this way the platelet accumulation and thrombus formation in the lumen surface of the vessels. Then, these grafts were implanted as carotid interposition grafts in rabbits and remained for a time period of 3 months [206]. The results of this study were impressive, indicating the long-term patency (over 12 weeks) of the hUAs treated with PSS/PAH film. Furthermore, successful cell invasion of PECAM^+^ ECs and α-SMA^+^ SMCs was evident in tunica intima and media, respectively [206]. Minimum intimal hyperplasia was reported in these grafts, which were mainly exerted through collagen production from SMCs [206]. Several months later, Gui et al. [208] evaluated a novel decellularization protocol in hUAs. In this study, a series of important experiments were performed, obtaining valuable information regarding the utilization of the hUAs as SDVGs [208]. Furthermore, the decellularized hUAs were implanted as abdominal interposition grafts in nude rats. After 8 weeks of implantation, thrombus formation was observed in the vascular grafts. Despite this drawback, decellularized hUAs sustained the blood flow and finally, the vessel did not rupture [208]. Several years later, the comprehensive proteomic analysis combined with histological data in native and decellularized hUAs was performed [209]. Until now, several researchers have evaluated the possibility of using the hUAs as transplants [206,207,208,209,210,211]. In 2020, our group showed that the decellularized hUA can be successfully vitrified and stored at −196 °C over a long time period [172]. Specifically, vitrified (decellularized) hUAs retained the ECM structure after 2 years of storage in liquid nitrogen. Furthermore, the vitrified grafts were used for common carotid bypass grafting in porcine animal models and remained for a time period of 1 month. Although the occurrence of platelet aggregation and thrombus formation was observed, vitrified hUAs were successfully in vivo remodeled [172]. The grafts were recellularized by the host’s VSMCs, and due to the blood flow stress-strain forces, increased production of elastin fibers was occurred [172]. By the time that this publication is prepared, another work from our group is focused on the biomechanical and proteomic characteristics of the decellularized hUAs [212]. The proteomic results have been deposited to the ProteomeXchange Consortium with the dataset identifier PXD020187 (https://www.ebi.ac.uk/pride/) and are currently publicly available. In this study, a rapid decellularization protocol was effectively applied in hUAs. No cellular or nuclear remnants were evident, while at the same time the proteomic and biomechanical analysis showed the preservation of key ECM structural proteins and mechanical characteristics of the hUAs, respectively [212].

HUAs may represent a better source for the development of SDVGs compared to hUVs. However, extended validation experiments to better determine the stability and functionality of these grafts should be performed. The future goal will be the successful recellularization with ECs/VSMCs and implantation to large animal models for longer time periods to acquire more valuable data regarding the possible application of hUAs as SDVGs.

### 4.5. In Vivo Performance of Decellularized and Cellularized SDVGs

Both decellularized and cellularized SDVGs have been tested in a wide series of experiments, including the evaluation of in vivo performance and biocompatibility [193,216]. It has been shown that decellularized SDVGs lack proper function and are characterized by a high probability of thrombus formation and graft failure [2]. Initially, the exposed collagen, located in the lumen side of the acellular SDVGs, triggers the platelets to aggregate [217]. The first step of this process involves the binding of the soluble form of von Willebrand factor (vWF) with the exposed collagen. Then, and upon vessel exposure to increased shear stress, the platelets are stimulated, leading to large aggregations development through the interaction between platelet glycoprotein (GP) Ib-V-IX receptor and vWF [218]. Furthermore, additional platelet receptors, including GPVI and α_2_β_1_, offer more stability to the developing thrombus [219]. Besides, the exposed collagen, fibronectin, and laminin assist in the development of thrombus, through the interaction with platelets’ integrins α_5_β_1_ and α_6_β_1_, respectively [217]. Furthermore, VSMCs contribute significantly to vessel functions. VSMCs is a specific smooth muscle cell subset, located in the tunica media of the vessel wall, responsible for vasoconstriction and vasorelaxation [39]. In these processes, the role of ECs in the regulation of vascular tone is very important. Upon stimulation of ECs, due to high shear stress, the nitric oxide (NO) synthase is activated and increases the NO production, which can cause vasorelaxation. In addition, VSMCs produce high amounts of ECM proteins, including collagen, elastin, and fibronectin, contributing to the regeneration of the injured vessels. An additional important function of VSMCs is the ability to retain the circumferential orientation of the collagen and elastin fibers in the vessel wall [61]. It has been shown that the removal of VSMCs during the decellularization process could alter the alignment of the collagen and elastin fibers. The presence of uncrimped collagen and elastin fibers in decellularized vascular grafts can induce significant alteration to their biomechanical properties, resulting in mismatch compliance between resident and transplanted vessels [212].

On the other hand, cellularized engineered SDVGs are conduits with improved properties, which may result in a more favorable outcome upon implantation. For this purpose, vessel bioreactors such as pulsatile flow or dynamic culture systems, are currently used, which can result in vessel production with uniform coverage of ECs and VSMCs. It has been shown in the literature that recellularized TEVGs are characterized by a lower risk for thrombus formation and graft rejection compared to non-cellularized TEVGs. Zhou et al. [220] reported low neo-intimal formation in recellularized vessels, which were obtained from decellularized canine vessels. In addition, Kaushal et al. [221] and Ma et al. [222] showed that the presence of ECs and VSMCs in the manufactured TEVGs contributed to neo-intimal and neo-medial reconstitution. Row et al. [223] managed to trace the cells used for the recellularization of TEVGs postoperatively. The recellularized TEVGs were implanted as interposition grafts into the coronary artery in female sheep for a time period of three months. In this set of experiments, it was observed that donor cells (used for the recellularization) were gradually substituted by the host cells. Importantly, donor cells represented only 17% and 8% of the total cells after 1 and 8 months postoperatively. Furthermore, no T or B cells were evident in the implanted vessels, indicating that the recellularized vessel favors no immune response [223].

Considering the above data, recellularized TEVGs have greater possibilities to avoid neo-intima formation, thrombus development, and graft failure, and are superior to decellularized vascular grafts. Nevertheless, greater effort regarding the recellularization process must be performed by the research groups worldwide to produce properly functional TEVGs.

## 5. Manufacturing Methods for the Development of SDVGs

Globally, the increased demand for vascular grafts requires the production of readily available functional transplants, although their large-scale production is a quite challenging task. Currently, there is a wide variety of SDVG manufacturing methods, including tissue-engineering by self-assembly (TESA), electrospinning, and bioprinting [224,225,226]. Since the first attempts of Weinberg, Bell, and Rosenberg in manufacturing vessel conduits, vascular graft production has been evolved and represents a quite interesting and interdisciplinary research field of the 21st century [33,176]. As has been proposed by Langer and Vaccanti, tissue engineering aims to the production of scaffolds or matrices in order to replace or remove the damaged tissues [227]. In the same way, the development of vascular grafts is characterized by the same principles. A number of different scientific areas must be combined to properly produce a vascular graft, which will replace the damaged one. Nowadays, the production and transplantation of LDVGs have been proven efficient based on the performed clinical trials [1]. On the other hand, the production of fully functional SDVGs is still under the developmental stage, needing further exploration. A few companies have achieved significant progress in the production of readily available SDVGs. Among them, Cryolife, Artegraft, Integra, and Gore are providing ready to use vascular grafts, derived mostly from in-house production (synthetic grafts), animal origin (bovine and porcine vessels), and cadaveric donors (human vessels). However, the clinical utility of vessel transplants requires also the evaluation of alternative methods for the production of vascular grafts. The natural ECM structure and its key mechanical properties are difficult to be reproduced with the aforementioned methods, but significant progress to this direction day by day is made.

### 5.1. TESA Approach

The TESA approach was developed by the pioneer L’Heureux and aimed at the production of vascular grafts utilizing cell sheets [224]. To achieve this outcome, no supporting vascular scaffolds are required.

**Figure 3:**
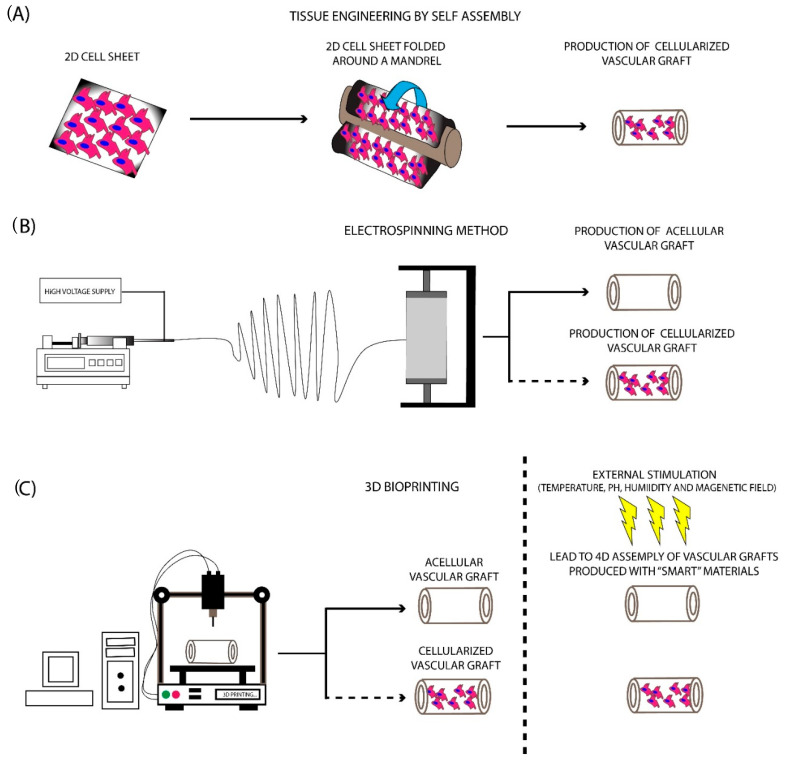
Fabrication methods for the production of SDVGs. (**A**) Production of SDVGs with the originally proposed method of L’Heureux et al. In this method, the production of SDVGs was relied on the self-assembly of cell sheets using a tubular mandrel. (**B**) Production of SDVGs with the electrospinning method. This methodology can produce complicated extracellular matrices (ECMs). In addition, combination with cellular populations can lead to the development of cellularized structures. (**C**) Production of SDVGs with the bioprinting method. Bioprinting offers the potential for the production of either acellular or cellularized complicated structures. Moreover, when used “smart” materials in the production process, the final product can assembly on the desired structure upon external stimulation (e.g., temperature, pH, humidity, and magnetic field).

The basic concept has relied on the use of cell sheets containing fibroblasts, mesenchymal stromal cells (MSCs), ECs, and VSMCs, which were shaped around a mandrel to produce a tubular formation (Figure 3). Further maturation in the pulsatile bioreactor is required in order to vascular grafts to achieve the prerequisite burst pressure and overall mechanical properties [224,228]. The initial work of L’ Heureux et al. [229] involved the cultivation of SMCs and fibroblasts in a standard culture medium contained sodium ascorbate. One month later, the produced sheets were shaped with the use of a tubular mandrel. The same technique was applied for the production of the different layers of the vascular graft. The results of this study were impressive. Specifically, histological analysis revealed the proper localization of the cellular populations, while the burst pressure of the produced vascular conduit was more than 2500 mmHg [229]. Moreover, these vessels were implanted as femoral artery interposition grafts in canine animal models, withstood the blood flow, and met the fundamental requirements of a vascular graft. More experiments also were performed utilizing human cells for the development of vascular grafts and their testing in different animal models. The above results led to the performance of the first clinical trial with TESA-produced TEVGs [229].

Between 2004 and 2007, a multicenter clinical trial was performed in patients who followed hemodialysis [230]. In this study, the vascular grafts were produced from the patient’s own cells (fibroblasts and SMCs) utilizing the cell-sheet technique in the same way as previously has been described. The produced grafts were characterized by a mean burst pressure of 3512 mmHg and were used as AVF conduits. The patency rates of the grafts were 78% and 60% after the 1st and 6th months of implantation, respectively. The most observed complications involved the development of aneurism and lumen thrombosis. Despite these drawbacks, this study represents an initial step toward the clinical utility of TESA-produced vascular grafts [230].

### 5.2. Electrospinning

The electrospinning method comprises the first attempt to mimic the complex structure of natural ECM. This method was introduced in 1930, providing an economical solution for scaffold fabrication [231]. Nowadays, its use has been expanded, thus scaffolds for bone and cartilage regeneration can be manufactured efficiently. Electrospinning has relied on the production of nano- and microfibers derived from a viscoelastic solution, where a high electrostatic force is applied. More specifically, the material that will be electrospun is pumped at a slow rate, ending in a high voltage electrical field [34,232]. This, in turn, leads to charging the polymer material during its exit from the syringe, which results in the production of the Taylor cone. A narrow jet of liquid is generated from the Taylor cone, which is further collected to a specific set up, known as the collector (Figure 3). Finally, the production of a scaffold, characterized by adequate ECM structure and fine-tuning mechanical properties, is produced [2,34]. The formation of the produced fibers is affected by various parameters, which are specific for the material, used each time, including molecular weight, surface tension, density, and viscosity [233]. Except for those, other parameters that can affect the fiber composition mostly include the applied electrostatic field, temperature, humidity, and flow rate of the polymers [233].

The polymer materials used in the electrospinning approach could be either degradable or natural derived materials [2,34]. However, important differences between the different materials exist. In the past, degradable materials such as PLA, PGA, PCL, PU/silk fibroin have been used for the production of scaffolds and specifically tubular conduits utilizing the electrospinning approach [2,34]. Vascular grafts have also been fabricated with the electrospinning method. Importantly, the proper combination of PLGA with collagen type I and elastin can improve the mechanical properties of the produced scaffolds, and their use is preferred for the production of electrospun blood vessels [234]. Moreover, it has been shown that the addition of naturally derived materials, such as collagen, gelatin, and fibronectin, may provide more RGD-binding sites, thus improving the cellular functions, like adhesion, growth, and differentiation [235].

In the context of electrospun tubular scaffold application, both acellular and cellularized conduits have been evaluated. Wise et al. [144] developed a tubular scaffold consisted of tropoelastin and PCL with the electrospinning method. The produced scaffold was characterized by similar biomechanical properties as the internal mammary artery (IMA). Further investigation involved the implantation of the acellular conduit in animal models [144]. Furthermore, the biomechanical analysis was performed in electrospun vascular grafts pre- and post-implantation. Specifically, acellular electrospun vascular conduits were implanted as carotid artery interposition grafts in rats for a total period of 1 month. Histological analysis in the explants showed the successful recellularization of the vascular grafts with ECs. Moreover, the explanted electrospun vascular grafts were able to preserve the initial vessel morphology and characterized by similar biomechanical properties as the pre-implanted grafts. In this study, the successful accumulation of tropoelastin in PCL scaffolds was shown for the first time, resulting in the production of vascular grafts, which were characterized by impaired platelet adhesion and increased endothelialization [144]. Additionally, Soletti et al. [236] provided substantial evidence regarding the proper development and production of anti-thrombogenic vascular conduits. Soletti et al. [236] showed that the acellular poly(etherurethane urea) (PEUU) grafts coated with the non-thrombogenic 2-methacryloyloxyethyl phosphorylcholine copolymer showed better patency and mechanical properties compared to uncoated PEUU vascular grafts [236]. Unlike Wise et al. [144] and Soletti et al. [236], Min Ju et al. [237] managed to develop electrospun bilayer tubular scaffolds consisted of PCL and collagen type I. Then, ECs and SMCs obtained from female Dorper Cross Sheep were seeded onto the tubular scaffolds, followed by maturation in the pulsatile flow bioreactor. The seeded vascular grafts were implanted as carotid artery substitutes in the sheep model and remained for 6 months. The electrospun vascular grafts were remained patent and the histological analysis revealed the production of collagen, elastin, and glycosaminoglycans within 6 months of implantation [237]. This study provided valuable data regarding the production and application of the electrospun vascular grafts. Moreover, Du et al. [238] used the electrospinning method to fabricate a 3D vascular microenvironment. In this approach, immobilization of VEGF onto the electrospun tubular scaffold consisted of gradient chitosan and PCL nanofibers was performed. The controlled release of VEGF potentially can enhance the adhesion of ECs and SMCs and further promote their rapid proliferation. In this way, engineered SDVGs with improved anti-thrombogenic properties could be developed, leading to the avoidance of lumen occlusion and thrombus formation, a series of common manifestations which are presented several days after the vessel implantation [238]. Taking into consideration the above data, it was clearly shown that electrospinning could be applied for the efficient production of engineered SDVGs. The produced electrospun SDVGs could be successful in vitro seeded with cellular populations and maintain further their graft patency, mechanical properties, and vessel integrity over a long time period.

### 5.3. Three Dimensional (3D) Bioprinting

In the last decade, 3D printing technology has gained significant attention and has been utilized with great success in a wide range of applications [239]. Using this technology, complex structures and materials can be produced efficiently, thus can be further used by the scientific society. The evolution of printing technology is 3D bioprinting, which has currently been applied in various tissue engineering approaches [239]. 3D bioprinting can produce complex structures, utilizing non-degradable/degradable and naturally derived polymers [240]. The significant potential of this methodology is the production of ready to use transplantable scaffolds and tissues. Currently, the 3D bioprinting approaches such as inkjet, extrusion, and laser-assisted bioprinting are mostly used for the production of the majority of the scaffolds [240]. A great series of materials are compatible with the bioprinter applications, although the polymer materials are mostly preferred in comparison with the naturally derived materials [240,241]. The bioprinter materials can be distinguished into three categories: (a) fibrous materials, (b) powder materials, and (c) bioinks. The use of the starting material is dependent on the characteristics of the produced scaffold [240,241].

3D bioprinting approaches and the proper combination of the aforementioned materials have been successfully applied in the production of LDVGs and SDVGs. [240,242,243,244]. In this direction, Freeman et al. [245] presented for the first time a new approach for the development of SDVGs using a custom-made 3D bioprinter. In this study, gelatin and fibrinogen were properly combined, producing a bioink with good rheological and printability properties. The produced vascular graft provided a favorable ECM for cell attachment. However, comprehensive in vitro and in vivo evaluation is further needed to be performed [245]. Jia et al. [246], in their study, used a multilayer coaxial nozzle device to produce vascular grafts. Moreover, human umbilical vein endothelial cells (HUVECs) and MSCs were expanded and encapsulated in a gelatin methacryloyl (GelMA), sodium alginate, and 4-arm poly(ethylene glycol)-tetra-acrylate (PEGTA) based bioink. Using the current bioprinter set-up in combination with the developed bioink resulted in the printing of highly organized vascular structures. No sign of cytotoxicity was reported, and after a time period of 21 days, the cells filled the entire printed vascular grafts. To evaluate better the cell behavior into the vascular wall, immunofluorescence was performed, showing the positive expression of α-SMA and CD31, in MSCs and HUVECs, respectively [246].

### 5.4. Four-Dimensional (4D) Bioprinting

Next-generation bioprinting demands the use of materials capable of self-transform into a prerequisite shape in order to exert their key functional properties. This state-of-the-art approach is known as 4D bioprinting and has gained increased attention in the last decade by the entire scientific community [247]. 4D bioprinting uses the same materials as conventional 3D printing approaches [240]. The major difference between 3D and 4D bioprinting is that the latter exerts a “smart” behavior of the produced scaffolds [248]. 4D produced scaffolds are superior to the conventionally bioprinted scaffolds. The “smart” behavior corresponds to “materials that can change their physical or chemical properties in a control and functional manner upon exposure to an external stimulus” as has been referred to by Tamay et al. [240]. In this way, 4D printed materials upon exposure to external stimuli such as pH, heat, magnetic field, light, and humidity can adopt effectively different shapes, exhibiting different properties [249]. The above-mentioned factors are playing important role in scaffold’s shape-changing properties. There exists a great variety of materials that achieve shape-transformation in response to temperature stimuli. Thermoresponsive materials are the most commonly used in 4D bioprinting applications [250]. These materials can be distinguished into (a) shape memory polymers (SMP) and (b) responsive polymer solutions (RPS). The first category involves polymers consisting of two distinct components, the elastic segment which is characterized by high glass transition temperature (Tg_h_), and the switching segment, characterized by intermediate glass transition temperature (Tg_i_). When the applied temperature is above the Tg_h_, the produced scaffold adopts its permanent shape. On the other hand, when the temperature is between Tg_i_ and Tg_h_, the switching segment becomes soft, while the elastic segment resists any shape-changing [251]. Additionally, if the material is cooled below the Tg_h_, then the elastic segment cannot return to its initial shape and the produced scaffolds acquire its final definitive form. SMPs include mostly the poly(ε-caprolactone) dimethacrylate (PCLDMA), polycaprolactone triol (Ptriol), and poly(ether urethane) (PEU). These materials have been used mostly in applications such as bone and cartilage engineering. RPS is characterized by a critical solution temperature, where if the applied temperature is above the aforementioned temperature (critical solution temperature), the polymer chains are contracting and the overall solution is adopting a solid form [247].

Both hydrophobic and hydrophilic interactions are existing between the polymer chains. In addition, a change in temperature may affect the behavior and the interaction of the above polymer chains. This, in turn, leads to shrinkage or expansion, which is a characteristic of each polymer material. In this direction, a material with a critical solution temperature above 25°C, when implanted to a mammalian organism, would expand, acquiring its definitive form. Poly(N-isopropylacrylamide), poly(ethylene glycol), collagen, gelatin, and methylcellulose are some of the most used RPS [249].

Besides the temperature stimuli, materials that can respond to pH changes also can be widely applied in the clinical setting [247]. The initial structure of these materials is consisting of acidic or basic groups, which are the main players in proton exchange upon pH changes [252]. In this way, polymers consisting of acidic groups, when exposed to pH > 7, act as anionic compounds, while polymers with a basic group, exposed to pH < 7, act as cationic compounds. Therefore, these materials upon pH stimuli can acquire different structural and functional properties, including change in solubility, degradability, swelling, etc. [248]. Like thermoresponsive polymers, pH-responsive polymers also can be utilized in a wide range of applications. Indeed, different human body compartments are characterized by different pH in order to serve properly their initial function, including the gastrointestinal tract, stomach, small intestine, and different regions of the vascular system including the kidney vascular network. Additionally, many solid tumors induce pH changes upon their growth. In this way, pH-responsive polymers can act as DDS, delivering tumor-specific therapy, such as signaling and cell proliferation inhibitors or monoclonal antibodies [253]. Examples of the most commonly used materials in this category are poly(acrylic acid), poly(aspartic acid), poly(L-glutamic acid), and poly(histidine), which can be combined effectively with naturally derived materials such as collagen, gelatin, and chitosan [252].

Besides the aforementioned, other categories of responsive materials have also been manufactured. These categories mostly include the photoresponsive, magneto-responsive, and humidity-responsive materials. However, their potential use is limited and, therefore, further evaluation of their properties is clearly needed. Briefly, photoresponsive materials can change their structural and functional properties, including wettability, solubility, degradability upon photo-stimulation [240]. Considering this, polymer materials with photosensitive groups can be manufactured, where the produced scaffolds can swell or shrink when specific photo-stimulation is applied. The combination of magnetic particles with polymer materials results in the development of magneto-responsive materials. The most commonly used magnetic particles are iron (Fe), nickel (Ni), cobalt (Co), and their oxides [249] Currently, magneto-responsive materials have been used as targeted therapeutic vehicles, carrying anti-tumor drugs. On the other hand, significant adverse reactions may be induced by their use in living organisms [254]. It has been shown that magnetic particles with a size less than 50 nm are transportable through the biological matrix, which can further cause inflammation and cell death due to high reactive oxygen species (ROS) production, DNA damage, and cytochrome C release. In this category, materials such as Fe_3_O_4_/PCL, Fe_3_O_4/_poly (ethylene glycol diacrylate), PCL/iron doped hydroxyapatite (PCL/FeHA) are currently evaluated for their potent use in living systems [254]. Lastly, humidity responsive materials also have been proposed for their use in 4D bioprinting and the production of tissue-engineered scaffolds. Interestingly the change in humidity could result in shape-change modification, which can act as a driving force for movement. These materials have not received great attention from the scientific community due to their limited use. Humidity responsive materials include poly(ethylene glycol) diacrylate, cellulose, polyurethane, and their combinations [255].

The 4D bioprinting comprises an important evolution in the fabrication of tissue-engineered scaffolds. Vascular grafts can be developed with this next-generation approach. In this way, we can imagine the development of a 4D bioprinted vascular graft (with a large or small diameter), which can acquire its specific shape inside the living organism upon temperature stimulation. Moreover, changes in pH of the vascular network may stimulate the implanted vascular graft in a way either to acquire a different shape or to substantially release key therapeutic agents in order to reduce or even to reverse the occurred situation. In the future, “smart” telebiometrics vascular grafts will be plausible to be employed, which can detect the changes of human body conditions, like temperature, pH, osmolarity, and will be able to notify or even to reverse a health issue. Currently, the utilization of “smart” materials and the manufacturing of those scaffolds is under the developmental stage [247]. Therefore, no significant number of publications is currently existing, with the only exceptions of reviews and opinion articles in this field. In this way, the development of “smart” materials that can be used in vascular engineering is quite important, but further exploration of this research field is needed.

## 6. Concluding Remarks

Globally, there is an increasing demand for SDVGs, as they are employed primarily in cardiovascular reconstruction surgeries. Indeed, more than 400,000 bypass surgeries are performed each year [12,13,14,15]. Parameters such as the modern way of life, increased working hours, overall stress, lack of physical exercise, and smoking comprise important risk factors for CVD development [18,20]. From an economical point of view, CVD is also a serious burden for all countries; therefore, novel and better treatment options must be utilized [21]. In this direction, the production of SDVGs and their efficient application in patients suffering from disorders that are belonging to CVD could be an important alternative strategy. One of the applied therapeutic approaches that are currently followed is the replacement of damaged vessels with autologous vascular grafts, such as the SV, mammary artery, and others [208]. However, CVD can affect the entire circulatory system, therefore, less than 60% of the patients have suitable vessels. Moreover, when second vascular reconstruction is needed to be performed in the same patient, this percentage is lower than 15% [31]. Blood vessel compatibility is another important parameter that should have in mind. Compliance mismatch between native and implanted graft (mostly at the anastomosis site) could induce unfavorable results, including calcification initiation, intima hyperplasia, lumen occlusion, platelets aggregation, and thrombus formation [32]. To avoid the above manifestations and in order to the availability of the vascular grafts to be increased, alternative strategies must be explored. Currently, the fabrication of LDVGs is efficient, using the latest TE approaches; therefore, the utilization of these methods could be applied in SDVGs production [2]. Although the significant drawbacks which engineered SDVGs may present, the interest of the scientific society is increasing day by day, exploiting better strategies to improve further the development of those grafts. Nowadays, the production of the SDVGs relies on the use of synthetic (non-degradable/degradable), naturally derived materials, and decellularized ECM. These materials can be combined with state-of-the-art manufacturing approaches (TESA, electrospinning, bioprinting) to produce vascular grafts with improved properties [2,245]. In most of these approaches, cell seeding and maturation in bioreactors (mostly pulsatile flow bioreactors) are needed in order to produced vascular grafts to effectively cellularized and acquire the proper biomechanical properties. Additionally, the decellularization of tissues and organs is a very promising approach, especially for the development of SDVGs. Indeed, decellularization can efficiently be applied in vessels such as the umbilical arteries or vessels derived from cadaveric donors to produce properly defined SDVGs.

In vascular engineering, different cellular populations have been proposed such as ECs, VSMCs, and stem cells, derived mostly from the recipient, avoiding in this way any unfavorable immune response and possibly graft failure and rejection [34]. However, a great number of cellular populations are needed for tissue engineering approaches. Terminally differentiated cells such as ECs and VSMCs can be isolated from a vessel biopsy, although to reach cell numbers >10 × 10^7^ requires extended in vitro manipulation and cultivation. On the other hand, the use of stem cells such as Mesenchymal Stromal Cells (MSCs) may be a more feasible approach. MSCs initially were isolated from bone marrow aspirates, while currently other sources including the adipose tissue and stromal vascular fraction can be used [256,257]. MSCs is a heterogenic multipotent stem cell population derived from mesoderm, and capable to differentiate effectively to “chondrocytes”, “adipocytes”, and “osteocytes” [258]. Immunophenotypically, these cells express (>95%) CD73, CD90, and CD105, while lacking the expression (<3%) of CD34, CD45, and HLA-DR, as has been indicated by MSCs committee of the International Society of Cell and Gene Therapy (ISCT) [259]. MSCs can be easily in vitro handled, while their stemness (specific gene expression and protein production) can be retained for an increased number of passages (>P8). Another candidate stem cell population for vascular engineering may be the induced pluripotent stem cells (iPSCs). In 2006, for the first time, Shinya Yamanaka managed to gain the pluripotent state of terminally differentiated cells by introducing a set of specific genes, including OCT4, SOX3, KLF4, and C-MYC [260]. Currently, different strategies have been developed for the production of iPSCs, even avoiding the use of *C-MYC*, a known oncogene. The efficient differentiation of iPSCs into various cell populations such as neural cells, cardiomyocytes, hepatic cells, ECs, VSMCs, etc. has been demonstrated in literature [261,262]. However, this technology has not yet received FDA approval for human clinical use, and therefore their applicability is limited even in vascular engineering [263,264].

The cellularization of TEVGs comprises a crucial step in the manufacturing process. Indeed, it has been shown that cell-seeded vascular grafts are characterized by better integration properties in the recipient’s body [2]. In this way, properly cellularized vascular grafts can avoid the interaction with M1 macrophages, favoring in this way the attraction of M2 macrophages. M2 macrophages have been related to the tissue remodeling process, avoiding the activation of T and B cells [265]. Moreover, several research groups have observed the development of vasa vasorum, the responsible vessels for nutrient supplementation, into the transplanted vascular grafts, indicating its further proper adaptation by the studied living system [172,266].

The proper manufacturing of SDVGs is an important aspect, and for this purpose, specific evaluation tests must be performed before their final application. These tests include (a) histological analysis, (b) biochemical and DNA quantification, (c) cytotoxicity assay, (d) platelet adhesion assays, (e) biomechanical analysis, and (f) implantation in animal models in order to assess effectively functionality of the vascular grafts. The above processes represent the first line of evaluation tests that should be performed to assess the biocompatibility of the manufactured SDVGs. Furthermore, more tests need to be performed to properly define the produced TEVGs. Especially for the SDVGs, the performance of cytotoxicity and the platelet adhesion assays is of major importance. In contrast to LDVGs, manufactured SDVGs are characterized by an increased probability of platelet aggregation and thrombus formation. Moreover, ECs should be properly seeded in SDVGs to produce a functional endothelium; therefore, the establishment of a non-toxic vascular graft is highly recommended.

Taking into consideration the above information of this review, we can conclude that the production of SDVGs is requiring further improvement, which is performed by several research groups worldwide. Currently, the use of synthetic and decellularized vascular grafts has gained a significant advantage over other methods. Highly organized ECMs cannot be in detail reproduced with the bioprinting approaches. Indeed, additive manufacturing techniques such as 3D and 4D bioprinting are characterized by a few limitations. The inability of reproducing the highly organized structure of SDVGs may comprise the most significant drawback of the current approaches. SDVGs are characterized by a complex structure, where collagen, elastin, fibronectin, and other key ECM proteins have specific relation and orientation in the vascular wall, ensuring in this way the proper recellularization. Cellularization of the bioprinted vessel constructs may be related to improved biocompatibility and biomechanical properties. In order to produce highly organized constructs, crosslinking of the biomaterials, used in bioprinting approaches, is preferred. However, the use of fixative agents such as glutaraldehyde, can result in increased cytotoxicity and altered biomechanical properties to the produced vascular scaffolds [103,205]. Moreover, when naturally derived bioinks are used, crosslinking may hamper the in vivo remodeling process of the vascular graft, leading to unfavorable outcomes. Moreover, the manufacturing of vessel constructs with high resolution demands high-cost printing devices and experienced personnel. Besides these drawbacks, in the future, the quality of bioprinters and printed constructs will be improved, leading to a new era in SDVGs development. Besides the above limitations, the introduction of 3D and 4D printing approaches may represent a new era regarding vascular scaffold production [240,247].

In conclusion, SDVGs now can be robustly produced and can be used in personalized medicine. Each production step must be specifically evaluated and the overall process must be performed in compliance with Good Manufacturing Practices (GMPs) conditions in order to produce readily available safe and fully functional grafts for patients suffering from CVD.

## Figures and Tables

**Table 1:** Representative applications of tissue-engineered vascular grafts (TEVGs) derived from non-degradable polymers.

Material Composition	Application	Comments	Research Team
Dacron	In vitro	Successful EC seeding in Dacron vessel conduits using either collagen-coated Dacron or fibronectin-coating ePTFE grafts	Sugawara et al. [65]
Dacron	In vitro	Coating of Dacron-based vascular graft with polyurethane. Increased porosity to the inner surface of the graft. Improved cell attachment properties	Phaneuf et al. [66]
ePTFE	Implantation in rabbits	ePTFE grafts were used as carotid artery interposition grafts, Good patency rate after 28 days of implantation, Successful endothelialization	Hytοnen et al. [67]
ePTFE	In vitro	Isolation of porcine ECs from jungular vein Successful endothelialization of ePTFE grafts Development of a bio-hybrid scaffold for vascular applications	Mall et al. [68]
ePTFE	Implantation in distal infrarenal aorta of rabbits	Development of ammonia plasma modified grafts Improved endothelialization of graft’s inner surface.	Sipehia et al. [69]
ePTFE	In vitro and in vivo evaluation	Development of polyurethane/polyurethane film Improved antiplatelet properties Lower hemolysis and no cytotoxicity (in vitro) Better biocompatibility, no occlusion, and successful endothelialization	Zhang et al. [70]
Dacron and ePTFE	In vitro	Immobilization of heparin, collagen, laminin, prostaglandin E1 (PGE1) Reduction of fibrinogen adsorption, and platelets deposition. Improved biocompatibility properties of both grafts	Chandy et al. [71]
Dacron and ePTFE	Implantation in mongrel dogs	Thrombus formation was reported 3 and 4 weeks postoperatively in ePTFE grafts. Patency rate of ePTFE grafts drop from 66% (3 weeks) to 33% (4 weeks) Patency rate of Dacron grafts changed from 55% (3 weeks) to 44% (4 weeks) ECs seeded grafts presented better patency rates and no graft occlusion due to thrombus formation. All animals received antiplatelet treatment	Hikro et al. [72]

**Table 2:** Representative applications of TEVGs derived from degradable polymers.

Material Composition	Application	Comments	Research Team
PCL	In vitro	Production of electrospun PCL SDVGs Modified surface with polyethyleneimine and heparin Prolonged anticoagulant action of the modified SDVGs Mild inflammation reaction (when implanted subcutaneously) May be characterized by great long-term patency. Future plan, implantation to animal models	Wang et al. [77]
PCL	Implantation in sheep	Thrombosis formation in the control group Good patency rate of PCL SDVGs (50% after 1st year of implantation)	Antonova et al. [80]
PCL	Implantation in mice	Acellular electrospun PCL-derived vascular grafts implanted as a carotid interposition graft Successful recellularization by host’s cells Complete endothelium formation within 28 days	Chan et al. [81]
PCL and PU	In vitro	Production of endothelialized SDVGs Good Biomechanical properties No significant differences in hemocompatibility between non-endothelialized and endonthelialized SDVGs	Mervado—Pagan et al. [82]
PGS	In vitro	Minimal platelet adhesion in the produced vascular graft No cytotoxicity to erythrocytes	Liu et al. [83] Motlagh et al. [84]
PLA	Implantation into rats	Antithrombogenic properties of MSCs Successful in vivo remodeling process Improved patency rate and no graft occlusion in BM-MSCs seeded vascular grafts	Hashi et al. [85]
PGA	In vitro	PGA derived vascular graft, seeded with VSMCs Maturation in a pulsatile flow bioreactor for 8 weeks Improved biomechanical properties (burst pressure 2150 mmHg)	Niklason et al. [86]
PGA	Implantation in baboons, canine	Implantation in baboons as arteriovenous conduits Implantation in canines as coronary artery interposition graft. Recellularization of PGA vascular graft with ECs. No aneurysm formation was reported Good patency rate in the majority of the vascular grafts after 1, 3, and 6 months in both animal models. Recellularization with host’s VSMCs and ECs	Dahl et al. [87]
PGA	In vitro and in vivo	Recellularization of PGA vascular graft with ECs and maturation in a pulsatile flow bioreactor ECs and induced pluripotent stem cells (iPSCs) in vascular tissue engineering	Gui and Niklason. [88]
PGA	Human Use	Recellularization of PGA vascular grafts with human ECs obtained from cadaveric donors Implanted in 59 patients as arteriovenous graft Improved patency rate compared to ePTFE grafts.	Lawson et al. [79]

**Table 3:** Representative applications of TEVGs derived from biopolymers.

Material Composition	Application	Comments	Research Team
Fibrin	In vitro	Combination of human dermal fibroblasts with vascular graft derived from fibrin gel Successful cell migration and collagen deposition Low biomechanical properties (burst pressure 543 mmHg)	Huyhn et al. [131]
Fibrin	In vivo	Fabrication of fibrin-based vascular graft Maturation of the graft in a pulsatile flow-stretch bioreactor Significant biomechanical properties (burst pressure 3164 ± 342 mmHg) corresponded to 99.8% of the reported value of human internal mammary artery Implantation as arteriovenous graft in olive male baboons The majority of the grafts remained patent for 6 months. Successful repopulation by host’s cells	Syedain et al. [132]
Fibrin	In vivo	Production of fibrin-based vascular grafts, seeded with ovine dermal fibroblasts. Implantation of the grafts as pulmonary artery replacements in Dorset lamps Implanted grafts were characterized by physiological strength and stiffness, complete lumen endothelialization, and repopulation by SMCs The lamps exhibited somatic growth and normal physiological function for nearly one year.	Syedain et al. [133]
Fibrin, collagen, collagen-fibrin	In vitro	Collagen and collagen fibrin vascular grafts share common biomechanical properties Fibrin-based vascular grafts are characterized by lower biomechanical properties than the above grafts SMCs proliferated equally in all vascular scaffolds	Cummings et al. [134]
Hyaluronan	In vitro	Addition of sodium ascorbate to hyaluronan-based vascular grafts Improvement in SMC proliferation and cell viability. Well organized ECM and good biomechanical properties	Arrigoni et al. [135]
Silk	In vivo (Implantation into Sprague-Dawley rats as abdominal aorta graft)	Better patency rate after 1 year of implantation, compared to ePTFE graft ECs and SMCs proliferation into the grafts within a short time after the implantation Good ECM organization and in vivo remodeling properties (inner and media layer) Observation of vasa vasorum	Enomoto et al. [113]
Silk	In vivo	Silk-based vascular grafts have equal mechanical properties as the rat abdominal aorta. Low platelet adhesion High proliferation potential of silk-based vascular grafts seeded with HUVECs and SMCs Vascular remodeling after implantation experiments in rats	Lovett et al. [136]
Collagen	In vivo	Development of collagen-based vascular grafts with burst pressure 1313 mmHg Endothelialization of collagen tubes after implantation in femoral artery of rats	Li et al. [137]
Chitosan	In vitro	Development of chitosan (2% *w*/*v*) vascular graft Burst pressure over 4000 mmHg Successful seeding with VSMCs obtained from rabbit aorta	Zhang et al. [138]

**Table 4:** Representative applications of TEVGs derived from hybrid materials.

Material Composition	Application	Comments	Research Team
PCL/collagen	In vivo	Development of hybrid scaffold with electrospinning method. Applied in aortoiliac bypass in rabbits, the graft remained for 1 month. Minimal cellular infiltration in the implanted vascular graft. Patency rate was 87.5% after 1 month of implantation	Tillman et al. [143]
PET/PU/PCL	In vitro and In vivo	Development of an electrospun triad-hybrid graft with an inner diameter of 5 mm. Burst pressure over 1689 mmHg Successful cell seeding and proliferation as it was indicated by the MTT assay Moderate immune reaction was observed after subcutaneous implantation in rats	Jirofti et al. [150]
PU/PET	In vitro	Development of PU/PET SDVGs with the electrospinning method Comparable biomechanical properties with native veins and arteries	Khodadoust et al. [151]
PU/PCL	In vitro	No cytotoxic PU/PCL vascular graft Successful seeded and proliferation of fibroblasts and ECs, as it was indicated by the MTT assay Confirmation of cell adhesion by SEM analysis	Nguyen et al. [152]
Gelatin/vinyl acetate	In vitro	Development of electrospun gelatin/vinyl acetate vascular grafts/ SMCs are used for seeding applications. Well organized ECM, accompanied by good biomechanical properties	Thomas and Nair et al. [148]
PCL and PU/collagen	In vivo	Electrospun PCL and PU/collagen vascular grafts were implanted as femoral artery interposition grafts in canines The grafts remained patent for 8 weeks Infiltration by ECs resulted in endothelium development	Lu et al. [153]
PCL/elastin	In vivo	Electrospun PCL/elastin vascular grafts were implanted as carotid arteries bypass grafts in rabbits The hybrid vascular graft was characterized by good biomechanical properties (tensile strength and Young’s Elastic Modulus) Low platelet attachment Preservation of biomechanical properties after implantation	Wise et al. [144]

**Table 5:** Summary of representative studies toward decellularized animal-derived vascular grafts.

Material Composition	Application	Comments	Research Team
Bovine carotid artery	In vitro	Decellularization of bovine carotid arteries with 1% *w*/*v* SD, 1% *w*/*v* CHAPS, 1% *v*/*v* Triton X-100 or 0.1% SDS Successful decellularization of carotid arteries Preservation of ECM structure Good biomechanical properties	Daugs et al. [186]
Ovine carotid artery	In vitro	Decellularization of carotid arteries with 1% *w*/*v* SDS, 0.05% *v*/*v* Trypsin, 0.02% EDTA Histological analysis with H&E, Masson’s Trichrome, and Verhoeff van Gieson revealed the preservation of ECM structure. Successful seeding and recellularization with MSCs	Mancuso et al. [187]
SIS	In vivo	Development of a vascular graft utilizing porcine SIS Implantation as a carotid artery interposition graft Functional comparison with autogenous saphenous vein No aneurism formation was found in both grafts. Equal patency rates between the two grafts	Sandusky et al. [188]
Bovine ureter	In vivo	Decellularized based on a patented process Comparison between ePTFE and decellularized bovine ureter. Applied as arteriovenous conduits Enrolled 60 patients No significant advantage of decellularized bovine ureter compared to ePTFE as AVF	Chemla and Morsy [189]
Bovine mesenteric vein	In vivo	Bovine mesenteric vein (MVB) evaluated as a vascular graft in hemodialysis Compared with ePTFE vascular graft Better patency rates of MVB than ePTFE graft (12 months was 35.6% for MVB versus 28.4% synthetic grafts. At 24 months, secondary patency was 60.3% MVB, 42.9% synthetic) Superior vascular graft compared to ePTFE grafts	Katzman et al. [190]
Canine carotid artery	In vivo	Decellularization of canine carotid arteries with 0.5% *v*/*v* Triton X-100, 0.05% *v*/*v* ammonium hydroxide Seeded with bone marrow MSCs derived from canine animal models Seeded grafts were implanted as carotid arteries interposition grafts Comparable suture retention strength between native and decellularized carotid arteries Successful in vivo remodeling after implantation, collagen and elastin production	Cho et al. [191]

**Table 6:** Summary of representative studies toward decellularized human-derived vascular grafts.

Material Composition	Application	Comments	Research Team
Cadaveric femoral vein	In vivo (large-scale clinical trial)	Commercially available decellularized human femoral vein (Synergraft^®^) Applied as allograft for Hemodialysis Comparison between Synergraft^®^, Cryovein and ePTFE grafts Impaired patency rate of human allografts compared to ePTFE grafts Aneurism formation observed in human allografts Human allografts cost 5 times more than ePTFE grafts Ethical concerns	Madden et al. [213]
Iliac vein	In vivo (Proof of concept study)	Decellularization of iliac vein with 1% *v*/*v* Triton X-100, 1% *v*/*v* tri-n-butyl phosphate, and 4 mg/L deoxyribonuclease Evaluation of presence of HLA class I and I antigens Recellularization with patient’s ECs and SMCs Vessel implantation After 1st year of implantation, the graft was occluded and a new surgical operation was performed. The second recellularized vascular graft remained patent. No need for immunosuppressive agents	Olausson et al. [195]
HUV	In vivo (large-scale clinical trial)	Stabilized hUV applied in femoropopliteal bypass grafting in 171 patients 6% of the patients died within the 1st year The patency rate was 65% and 50% within the first and fifth year, respectively.	Jarrett and Mahood [214]
HUV	In vitro	HUV denudation either with 0.1% *w*/*v* collagenase, hypotonic media, or with gentle gas stream for ECs dehydration Better denudation using stream of gas, according to histological, SEM and biomechanical results	Hoenika et al. [215]
HUA	In vitro and in vivo	Trypsin de-endothelialization of hUVs Development multilayer of PSS/PAH films Implantation as a carotid interposition graft in rabbits. Good patency over 12 weeks. Successful cell infiltration by PECAM^+^ ECs and α-SMA^+^ SMCs	Kerdjoudj et al. [206]
HUA	In vitro and in vivo	Decellularized hUAs with CHAPS, SDS, EDTA, and EGM-2 buffers Preservation of ECM structure while no cells were evident. Implantation as acellular abdominal interposition grafts. Thrombus formation, but the vessel lumen did not rupture	Gui et al. [208]
HUA	In vitro and in vivo	Decellularization of hUAs with CHAPS, SDS, and α-ΜΕΜ with 40% FBS. Good preservation of ECM structure, no cellular or nuclear material, good biomechanical properties. Implantation as common carotid interposition graft. Thrombus formation within 30 days after the implantation. In vivo remodeling of hUAs, elastic fibers production	Mallis et al. [172]
